# Influence of Sampling Strategies and Disease Prevalence on SARS-CoV-2 Detection Dynamics in Wastewater Surveillance

**DOI:** 10.3390/v18050583

**Published:** 2026-05-21

**Authors:** Siti Aishah Rashid, Mohd Ishtiaq Anasir, Fadly Syah Arsad, Nurul Farehah Shahrir, Khayri Azizi Kamel, Sakshaleni Rajendiran, Nurul Amalina Khairul Hasni, Mohamad Iqbal Mazeli, Yuvaneswary Veloo, Syahidiah Syed Abu Thahir, Wan Rozita Wan Mahiyuddin, Khor Bee Chin, Alijah Mohd Aris, Redzuan Zainudin, Rafiza Shaharudin, Raheel Nazakat

**Affiliations:** 1 Environmental Health Research Centre, Institute for Medical Research, National Institutes of Health, Ministry of Health Malaysia, Shah Alam 40170, Malaysia; farehah.shahrir@moh.gov.my (N.F.S.); sakshaleni@moh.gov.my (S.R.); drn.amalina@moh.gov.my (N.A.K.H.); dr.miqbal@moh.gov.my (M.I.M.); yuvanes@moh.gov.my (Y.V.); syahidiah@moh.gov.my (S.S.A.T.); redzgsc@gmail.com (R.Z.); rafiza.s@moh.gov.my (R.S.); raheel@moh.gov.my (R.N.); 2 Infectious Disease Research Centre, Institute for Medical Research, National Institutes of Health, Ministry of Health Malaysia, Shah Alam 40170, Malaysia; ishtiaq@moh.gov.my (M.I.A.); khayri.kamel@moh.gov.my (K.A.K.); 3 Kuala Lumpur & Putrajaya Health Department, Lembah Pantai District Health Office, Kuala Lumpur 50480, Malaysia; fadlysyah@moh.gov.my; 4 SEAMEO TROPMED Network Malaysia, Institute for Medical Research, National Institutes of Health, Ministry of Health Malaysia, Shah Alam 40170, Malaysia; rozita.wm@moh.gov.my; 5 Indah Water Konsortium Sdn. Bhd., Kuala Lumpur 60000, Malaysia; bckhor@iwk.com.my (K.B.C.); alijaha@iwk.com.my (A.M.A.)

**Keywords:** wastewater-based surveillance, SARS-CoV-2, digital PCR, sampling optimization, lag time, variant detection

## Abstract

Background: Wastewater-based surveillance (WBS) has emerged as a valuable tool for population-level monitoring of Severe Acute Respiratory Syndrome Coronavirus 2 (SARS-CoV-2) transmission, yet the interplay between sampling strategies and disease prevalence in shaping detection performance remains ambiguous. We investigated how grab and composite sampling influence SARS-CoV-2 ribonucleic acid (RNA) detection dynamics and predictive lag times across high- and low-prevalence communities in Selangor, Malaysia. Methods: A 28-week longitudinal study was conducted in Selangor, Malaysia, comparing grab and composite wastewater sampling in communities with high and low Coronavirus disease 2019 (COVID-19) prevalence. SARS-CoV-2 RNA in 348 samples was quantified using digital Reverse Transcription Polymerase Chain Reaction (RT-dPCR), and viral lineages were characterized by Nanopore sequencing. Detection sensitivity and lead times relative to reported cases were evaluated. Results: In low-prevalence settings, grab sampling showed higher detection sensitivity than composite sampling (92.0% vs. 70.0%), whereas both methods achieved similarly high detection in high-prevalence areas (>97.0%). Lag-time analysis indicated that grab sampling in high-prevalence settings was significantly associated with case trends at potential two-week lead (*p* = 0.024), while composite sampling in low-prevalence settings showed the strongest association at a potential one-week lead (*p* = 0.0022). Overall, lag structures varied by both sampling strategy and prevalence context. Both sampling approaches captured the replacement of Omicron sublineages (XBB.1.5, XBB.1.9.1, XBB.1.16) and identified additional circulating variants, including EG.5, that were not captured in the available clinical sequencing dataset during the same period. Conclusions: These findings reveal that local transmission intensity is associated with the utility of different sampling designs. Context-specific optimization of WBS sampling strategies enhances sensitivity, reduces detection lag, and strengthens early warning and genomic-tracking capacity in public health surveillance frameworks.

## 1. Introduction

Coronavirus disease 2019 (COVID-19) is a highly transmissible respiratory illness caused by severe acute respiratory syndrome coronavirus 2 (SARS-CoV-2). Since its initial emergence in Wuhan, Hubei Province, China, in December 2019, SARS-CoV-2 has spread rapidly to countries worldwide, which prompted the World Health Organization (WHO) to declare a Public Health Emergency of International Concern (PHEIC) on 30 January 2020 and a global pandemic on 11 March 2020 [1]. The COVID-19 pandemic exposed substantial gaps in public health preparedness both globally and in Malaysia, overwhelming healthcare systems, straining hospital and intensive-care capacity, and necessitating repeated movement restrictions that imposed significant societal and economic burdens [2,3,4]. A key lesson from the pandemic is the need for continuous, population-level surveillance systems that remain resilient despite limitations in clinical testing.

In this context, wastewater-based surveillance (WBS) has emerged as a robust and complementary tool to clinical surveillance, providing early, community-level indicators of transmission independent of testing uptake [5,6]. Because the virus is shed in feces and other excreta, its RNA can be reliably detected in wastewater, enabling population-wide monitoring [7,8,9]. Compared with clinical surveillance which is constrained by testing capacity, under-ascertainment, and variability in health-seeking behavior, WBS offers a proactive, non-invasive, and cost-efficient means of assessing transmission trends and circulating variants [10,11,12]. In Malaysia, the National Public Health Laboratory (NPHL) implemented a WBS program starting in June 2022, conducting monitoring across fourteen sentinel sites with integrated variant screening to guide the transition to endemic COVID-19. Similar national efforts in Norway [13], Denmark [14], Spain, and Italy [15] demonstrate the growing recognition of WBS as a complementary and resilient component of infectious disease surveillance.

Numerous studies have demonstrated that increases in wastewater viral ribonucleic acid (RNA) concentrations often precede clinical case notifications, with reported lead times typically ranging from four to ten days [16]. For example, SARS-CoV-2 was detected in Caracas, Venezuela, four to six days before official case reporting, while analyses in the United States observed median leads of 4–7.5 days between wastewater peaks and hospitalizations [7,17]. However, these lead times vary across settings, reflecting differences in viral shedding dynamics, epidemiological context, and methodological choices in sampling and analysis [18,19]. Optimizing WBS systems design and sampling strategies is therefore critical to maximize their accuracy and public health value.

Sampling strategies strongly influence SARS-CoV-2 detection in wastewater by affecting the ability to capture temporal variability in viral shedding. Grab samples, which represent a single time point, may be suitable in large, high-flow systems but can miss intermittent shedding, especially in small or low-flow catchments. In contrast, composite sampling integrates samples over time (e.g., 24 h), reducing variability and improving detection reliability [20]. Passive samplers, such as Moore swabs or tampons, further enhance detection by continuously accumulating viral particles over time [21].

Empirical studies support these differences. In Forest Grove, USA, grab samples performed similarly to composite samples in high-flow systems but showed greater variability and more false negatives in low-flow settings [22]. Other studies also report only moderate agreement between grab and composite samples, with composite sampling providing more representative viral estimates [23]. Passive sampling has demonstrated higher sensitivity, including detection of SARS-CoV-2 in samples that were negative by grab or composite methods [21]. Overall, these findings highlight that sampling design, together with flow conditions and infection prevalence, plays a key role in determining detection sensitivity and should be carefully optimized for effective wastewater surveillance.

WBS offers distinct advantages in the endemic phase by capturing aggregated viral shedding from entire catchments and buffering against under-ascertainment in clinical reporting. Yet, despite its rapid expansion globally, important methodological questions remain regarding how sampling design interacts with local transmission intensity to influence detection performance, particularly in low- and middle-income settings. The efficiency of WBS is shaped not only by analytical workflows but also by the choice of sampling strategy. Grab and composite sampling capture different temporal features of wastewater flow, and their relative performance may vary with underlying prevalence, shedding variability, and sewer system dynamics. However, these factors have yet to be fully elucidated. We hypothesize that the effectiveness of sampling strategies (grab vs. composite) is modulated by community prevalence, resulting in differential detection sensitivity and predictive lead times relationships.

To address this gap, the present study evaluates how sampling strategies and community prevalence jointly influence SARS-CoV-2 detection dynamics, lead time associations, and variant resolution within Malaysian wastewater systems. By integrating digital PCR quantification, temporal modeling, and Nanopore-based genomic surveillance, we provide an evidence-based framework to optimize sampling design for robust, context-specific WBS implementation in the endemic phase of COVID-19.

## 2. Materials and Methods

### 2.1. Setting and Study Population

**Figure 1:**
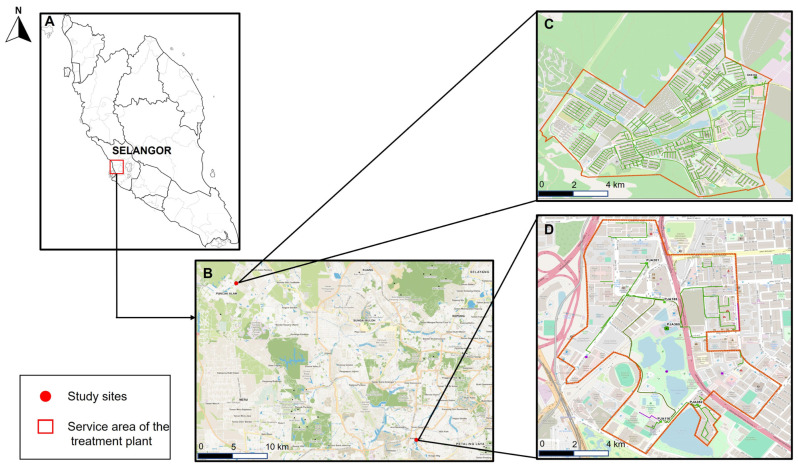
Location of the study sites and service areas of the treatment plants. (**A**) shows the location of Selangor within Peninsular Malaysia. (**B**) highlights the study areas in Puncak Alam and Petaling Jaya. (**C**,**D**) show detailed maps of the service areas for the treatment plants in Puncak Alam and Petaling Jaya, respectively, outlined in red.

The study was conducted in Selangor, Malaysia, in collaboration with the Indah Water Konsortium Sdn. Bhd. (IWK). Two wastewater treatment plants (WWTPs) were selected to represent areas with contrasting COVID-19 burden, operationalized according to Malaysia Ministry of Health (MOH) district zoning based on reported cases in the preceding 14 days. Catchments were categorized as higher prevalence (“red-zone equivalent”; ≥41 cases/14 days) or lower prevalence (“yellow/green-zone equivalent”; ≤40 cases/14 days) during the sampling period, and one WWTP from each category was selected [24,25,26]. Prevalence classification followed Ministry of Health (MOH) zoning criteria based on reported case counts over a 14-day period, consistent with national surveillance practices. Although not normalized to population size (e.g., cases per 100,000 population), this approach reflects operational public health definitions and enables comparison of contrasting transmission contexts. Accordingly, site selection was guided by the spatial distribution of COVID-19 cases across Selangor from February to August 2023, ensuring representation of both high- and low-prevalence communities. The selected WWTPs, located in Kelana Jaya (Petaling district) and Kuala Selangor district, serve a combined population equivalent exceeding 50,000 residents (Figure 1).

### 2.2. Sample Collection

The sampling procedure followed the protocol described by Rashid et al. (2025) [27], to monitor temporal trends of SARS-CoV-2 RNA levels in community WWTPs wastewater. Wastewater samples were collected from the influent stream of the selected WWTPs prior to treatment processes. Over a 28-week period (February to August 2023), wastewater samples were collected three times weekly (Monday to Wednesday) from two selected wastewater treatment plants (WWTPs). Two sampling approaches were employed: (a) composite sampling and (b) grab sampling. For composite sampling, 24 h flow-proportional samples (1 L) were collected using an ISCO automated sampler (Hoskin Scientific, Coquitlam, BC, Canada) operating every 4 h, with samples maintained at approximately 4 °C using dry ice. Grab samples (0.5 L) were collected during the morning peak flow period in sterile 1 L bottles, transported on ice, and stored at 4 °C until analysis. Subsequent processing, quantification, and sequencing were performed according to the analytical workflow outlined by Rashid et al. (2025) [27]. Although different total volumes were collected for grab (0.5 L) and composite (1 L) samples, a standardized aliquot (40 mL) was used for downstream processing to ensure comparability between sampling methods. Therefore, observed differences reflect sampling strategy rather than sample volume.

### 2.3. Sample Processing and RNA Extraction

Wastewater samples were processed following the protocol described by Rashid et al., (2025) [27]. Briefly, 40 mL of each sample was first treated with protease and incubated at room temperature for 30 min. The samples were then centrifuged at 5000× *g* for 10 min at room temperature to remove suspended solids and coarse particulate matter, and the resulting supernantant was used for total nucleic acid extraction (TNA) using the Maxwell^®^ Enviro Wastewater TNA Kit (Promega Corp., Madison, WI, USA). The clarified supernatant was transferred to a 250 mL tube (Corning-430776, Corning Inc., Corning, NY, USA), followed by the addition of binding buffers and isopropanol, and loaded onto a PureYield™ Midi Binding Column (Promega Corp.) using a vacuum manifold system for nucleic acid capture. The bound nucleic acid was washed and eluted with pre-warmed nuclease-free water. The extracted nucleic acid was further purified using the Maxwell^®^ RSC automated extraction system (Promega Corp.) and stored at −80 °C until quantification.

### 2.4. Quantification of SARS-CoV-2 RNA

The quantification of SARS-CoV-2 RNA was carried out using digital reverse transcriptase–polymerase chain reaction (RT-dPCR) with the GT-Digital SARS-CoV-2 Wastewater Surveillance Assay for QIAcuity^®^ (Qiagen, Hilden, Germany). The assay targeted the nucleocapsid (N1) gene regions of SARS-CoV-2. The PCR reactions were run on 26,000 24-well Nanoplates using the Qiagen QIAcuity 5-plex platform with thermal cycling conditions: (a) reverse transcription at 50 °C for 30 min, (b) enzyme activation at 95 °C for 2 min, and (c) 45 cycles of denaturation (95 °C for 10 s) and annealing/ extension steps (55 °C for 30 s). Pepper mild motile virus (PMMoV) was included as an internal control to verify the integrity of the fecal content. Positive and negative controls were incorporated in each run. Data were analyzed using QIAcuity Suite Software (v2.1.7.182), and concentrations were expressed as genome copies (GC) per L of reaction mixture. Normalization using fecal indicators (e.g., PMMoV) or flow-based parameters was not applied, as the study aimed to preserve direct comparability between sampling strategies and to avoid introducing additional variability associated with site-specific differences in fecal marker dynamics.

### 2.5. Targeted Amplicon Sequencing

Targeted amplicon sequencing was adapted from the protocol outlined by Ramachandran et al. (2022) [28], with minor modifications using the updated Oxford Nanopore Technologies (ONT) (Oxford, UK) V14 chemistry. In short, the extracted nucleic acid was treated with TURBO DNase (Invitrogen, Thermo Fisher Scientific, Waltham, Massachusetts, USA) to remove residual DNA and purified using RNAClean XP beads (Beckman Coulter, Brea, CA, USA). The purified RNA was reverse-transcribed for SARS-CoV-2 tiled amplicon generation using the VarSkip Short v2 with spike-in primers (VSS v2b) and the reagents included in the NEBNext ARTIC SARS-CoV-2 Companion Library Prep Kit (NEB, Ipswich, MA, USA). Amplicon sequencing libraries were prepared using the ONT Native Barcoding Kit 96 V14 (SQK-NBD114.96, Oxford Nanopore Technologies, Oxford, UK). Up to 23 samples and one negative control were barcoded in triplicate and pooled per library for each sequencing run on a MinION R10.4.1 flow cell. Sequencing was performed for 72 h using the ONT GridION platform with a high-accuracy (HAC) base-calling model via MinKNOW (v22.12.5) and Guppy (v6.4.6).

### 2.6. Data, Time Series and Bioinformatics Analysis

Samples were classified as positive when the N1 gene target was detected above the assay’s quantification limit. Positivity rates were calculated as the proportion of positive samples to the total number of samples within each sampling method (grab vs. composite) and area category (high vs. low prevalence). For temporal trend analysis, weekly viral RNA concentrations (copies/L) and confirmed COVID-19 cases were aggregated and smoothed using a seven-day moving average to capture short-term fluctuations and align wastewater signals with clinical outcomes. Descriptive lead–lag patterns observed in the time series plots were annotated for visual reference; statistical significance was evaluated separately using distributed lag model (DLM) and cross-correlation analyses.

Time series analysis was conducted to investigate the temporal relationship between SARS-CoV-2 RNA concentrations in wastewater and weekly reported COVID-19 cases over the 28-week study period. The data were aggregated by epidemiological week, and both viral concentration and case count variables were organized into a time series format to preserve temporal order.

A Distributed Lag Model (DLM) was fitted using lagged wastewater viral RNA concentrations from Lag 0 up to Lag 3 weeks as predictors of weekly COVID-19 case counts. The DLM framework was selected a priori because it is appropriate for evaluating delayed temporal associations between wastewater signals and epidemiological outcomes. To assess robustness, additional sensitivity analyses were conducted using generalized linear models (GLM) with a quasi-Poisson distribution and generalized additive models (GAM) with comparable lag structures. Analyses were based on an effective sample size of n = 26 weeks, accounting for the 3-week lag period. Model performance was evaluated using Multiple R^2^, Adjusted R^2^, and F-statistics. In instances where the Adjusted R^2^ was negative, the model was interpreted as having negligible predictive power. Out-of-sample validation was additionally performed using correlation between predicted and observed values and root mean square error (RMSE) to provide an indication of predictive stability.

Statistical inference was based on regression outputs, including parameter estimates, standard errors, and *p*-values for each lag. To ensure the reliability of our findings, we checked model assumptions using residual and autocorrelation (ACF) plots. We supplemented these visual diagnostics with formal hypothesis testing for serial correlation and performed Cross-Correlation Function (CCF) analysis as a sensitivity check to assess whether the lag structures identified by the regression models were consistent. Estimated lead times were interpreted as approximate temporal relationships rather than precise predictive intervals. Finally, we visualized the results by plotting raw weekly data against model-fitted values to assess the overall fit of the DLM. All analyses and visualizations were conducted in R, utilizing packages including dynlm for fitting distributed lag models and ggplot2 for graphical representation of the data. These analytical approaches were applied to test the hypothesized relationships between sampling strategy, community prevalence, and SARS-CoV-2 detection dynamics, including sensitivity and temporal lead times.

Bioinformatics analysis was performed using the Centre for Food Safety and Nutrition (CFSAN) Wastewater Analysis Pipeline (C-WAP) [29,30]. Fastq files from each sample were combined and processed with C-WAP, and mutations with at least 10× coverage were considered in the analysis. Samples with ≥60% genome coverage were further processed using Freyja (version 1.4.9) to estimate variant abundance and lineage composition relative to the Wuhan-Hu-1 reference genome (MN908947.3). Variant proportions were derived from median estimates using Freyja bootstrap function (nb = 10), with lineage classification based on the UShER barcode updated on 20 March 2023. Outputs were used to confirm SARS-CoV-2 detection and identify dominant circulating variants.

### 2.7. COVID-19 Genome Surveillance Datasets for Selangor, Malaysia

SARS-CoV-2 whole genome sequences from Malaysia were obtained through the data repository GISAID [31,32,33,34]. Data were filtered by location (Asia/Malaysia/Selangor) and date of collection (6 February 2023 to 31 August 2023). Sequences with unassigned lineages were excluded from this study. The findings of this study are based on metadata associated with 737 sequences available on GISAID up to 7 January 2026, via https://doi.org/10.55876/gis8.260107uh.

## 3. Results

### 3.1. Detection Sensitivity by Sampling Strategy and Prevalence

Between February and August 2023, a total of 348 wastewater samples were analyzed across two sites representing high- and low-prevalence catchments, as well as grab and composite sampling approaches. SARS-CoV-2 RNA was detected in 313 samples (90.0% overall positivity). In both study areas, a higher positivity rate was observed in samples collected through grab sampling compared to composite sampling. In low-prevalence areas, grab sampling exhibited a 22% higher positivity rate than composite sampling (92.0% vs. 70.1%), indicating greater detection capability when community viral shedding was low. Conversely, both sampling approaches achieved near-complete detection in high-prevalence areas (100% vs. 97.7%), indicating that both methods yield comparable results during periods of ongoing transmission (Table 1). This pattern is consistent with the proposed hypothesis that sampling performance varies according to underlying prevalence conditions.

### 3.2. Temporal Trends in Wastewater and Clinical Data

**Figure 2:**
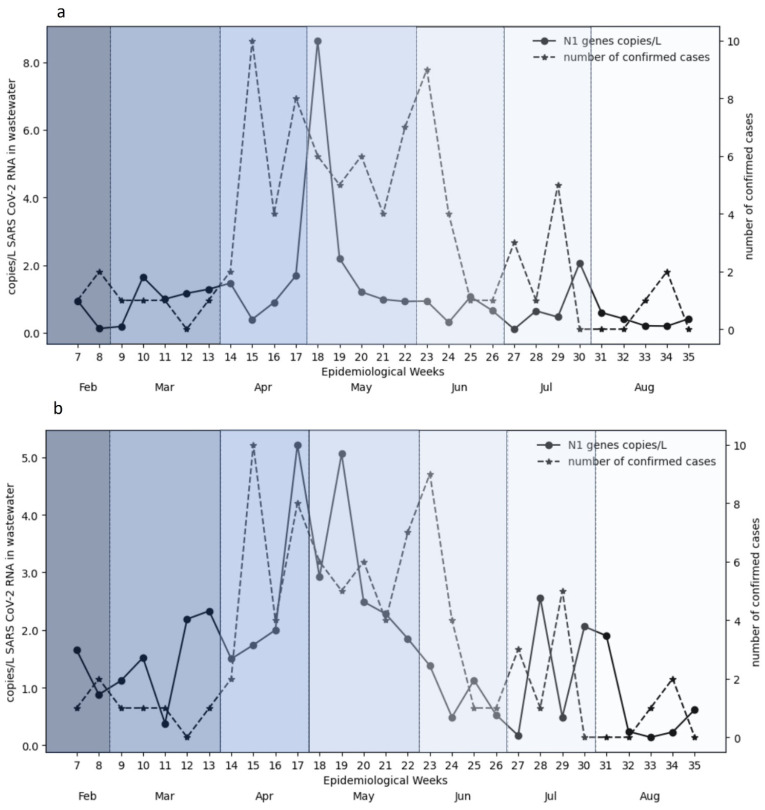
Temporal trends of SARS-CoV-2 RNA (N1 gene targets) in wastewater and confirmed COVID-19 cases in a high-prevalence area. Weekly observed values (points) and seven-day moving averages (lines) of SARS-CoV-2 RNA concentrations (copies/L) are shown for wastewater samples collected using (**a**) composite and (**b**) grab sampling methods. These trends are presented alongside confirmed COVID-19 cases in the corresponding service area over the seven-month study period (February–August 2023). Visual inspection suggests a temporal lead–lag pattern between wastewater signals and reported cases. Background shading indicates monthly intervals. The smoothed lines are provided for descriptive visualization; uncertainty intervals are not shown. Statistical evaluation of these relationships is provided in Section 3.3.

Time series analysis revealed that peaks in wastewater viral RNA concentrations typically preceded increases in reported COVID-19 cases. Reporting of COVID-19 cases was markedly lower during the study period compared to the pandemic years. SARS-CoV-2 viral loads measured at both WWTPs were plotted and compared with the number of active clinical cases reported by health authorities for selected coverage areas in the Petaling and Kuala Selangor districts. Smoothed seven-day moving averages showed strong temporal coherence between wastewater viral loads and case counts, with wastewater consistently serving as a leading indicator (Figure 1 and Figure 2). These trends support the predictive capacity of WBS, even under reduced clinical testing and case reporting volumes.

#### 3.2.1. High-Prevalence Area

At the high-prevalence site (Figure 2), SARS-CoV-2 RNA concentrations in wastewater (blue line) peaked sharply during epidemiological week 17–19 from mid-April to early May. Subsequent smaller surges were observed in epidemiological week 28 to week 30, from mid-July to early August 2023. Composite samples (Figure 2a) displayed sharper and more acute peaks, reaching over 8 copies/L (Figure 2a), while grab samples exhibited more frequent and broader peaks, reaching over 5 copies/L (Figure 2b), indicating a more integrated temporal trend with higher temporal variability. Comparing the wastewater viral concentration with concurrent clinical cases (orange line) in the same time line, the confirmed COVID-19 cases showed a lagged peak approximately four weeks later in week 23, following a sharp peak between week 17–19 for both composite and grab samples. However, the grab samples revealed a clearer trend, with peaks in viral concentrations from the wastewater consistently followed by corresponding peaks in clinical cases at a much shorter interval, which can be observed during both major and minor surges. The overall graph shows that there is a temporal pattern between the viral concentrations in wastewater and confirmed clinical cases within the study period.

#### 3.2.2. Low Prevalence Area

**Figure 3:**
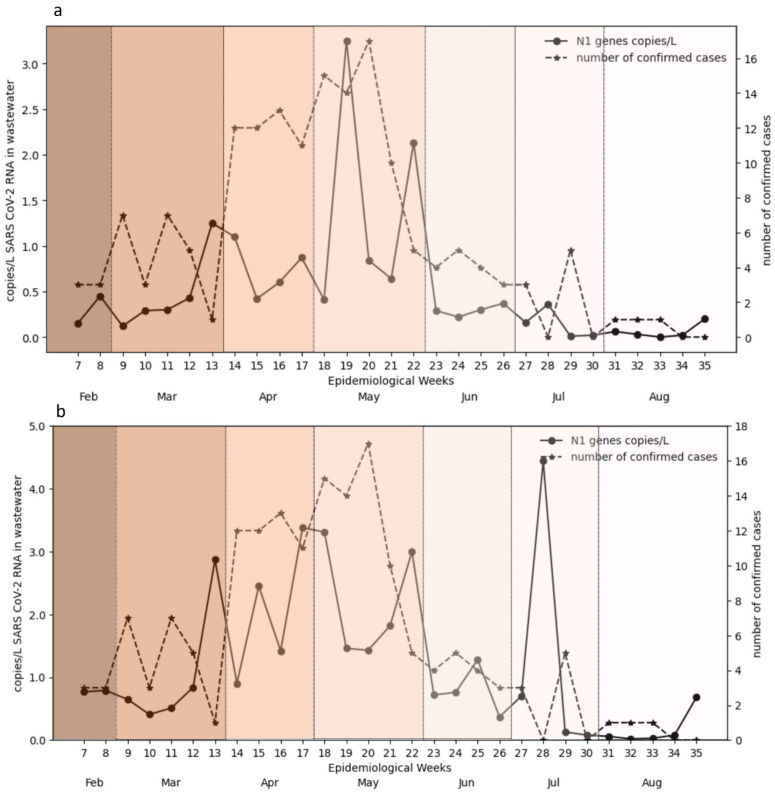
Temporal trends of SARS-CoV-2 RNA (N1 gene targets) in wastewater and confirmed COVID-19 cases in a low-prevalence area. Weekly observed values (points) and seven-day moving averages (lines) of SARS-CoV-2 RNA concentrations (copies/L) are shown for wastewater samples collected using (**a**) composite and (**b**) grab sampling methods. These trends are presented alongside confirmed COVID-19 cases in the corresponding service area over the study period (February–August 2023). Visual inspection suggests a consistent temporal lead–lag pattern between wastewater signals and reported cases across both sampling approaches. Background shading indicates monthly intervals. The smoothed lines are provided for descriptive visualization; uncertainty intervals are not shown. Formal statistical analysis is presented in Section 3.3.

In the low prevalence area (Figure 3), an initial period of low wastewater viral gene levels (blue line) was observed between weeks seven and ten, in both composite and grab samples (Figure 3a,b). Both sampling methods showed a marked increase from weeks 11 to 15. For composite samples (Figure 3a), this increase was followed by a subsequent decline before reaching another peak between week 18–20. In the case of grab samples (Figure 3b), multiple peaks in viral load were observed from week 11 (April) to week 23 (May), with a subsequent decreasing trend up to week 26. The highest values for grab samples were ultimately recorded during weeks 27 and 28, reaching nearly 4.5 copies/L.

Regarding confirmed COVID-19 cases in the low prevalence area, an initial low number of cases was observed from mid-February to early March 2023 (EW 7–13). A significant increase in cases was observed from early April 2023, and peaked around mid-April (reaching 10–12 cases). For both grab and composite sampling in these areas, multiple peaks in wastewater viral loads were followed by corresponding peaks in clinical cases with a one week-lead. Another notable surge in confirmed cases occurred around mid-May 2023 (reaching 16–17 cases), closely mirroring the peak in composite water samples. Following this, confirmed cases generally decreased, showing smaller peaks around early June to mid to late July 2023. Toward the end of the study period (early to late August 2023), confirmed cases remained low or showed minor fluctuations, largely mirroring the low wastewater viral concentrations.

### 3.3. Lag-Time Modeling and Predictive Associations

To test the hypothesized relationship between sampling strategy, community prevalence, and detection dynamics, we evaluated lag-specific associations between wastewater viral concentrations and reported COVID-19 cases using DLM as the primary model, supplemented by GLM and GAM sensitivity analyses and out-of-sample validation. In epidemiological surveillance, early detection and rapid information processing are critical for effective public health response. We analyzed the temporal features of SARS-CoV-2 WBS, focusing on peak timing and predictive associations in both high- and low-prevalence settings using an effective sample size of n = 26 weeks per model.

In the high-prevalence setting, grab sampling demonstrated a significant lagged association with weekly reported COVID-19 cases. In the DLM, Lag 2 was significantly associated with case counts (estimate = 0.238, *p* = 0.024; adjusted R^2^ = 0.26), suggesting an approximate two-week lead relationship. This pattern was consistent across modeling approaches, with GLM confirming the significance of Lag 2 (*p* = 0.025) and GAM indicating a similar lag structure with moderate explanatory capacity. Validation analysis indicated moderate predictive stability (r = 0.72; RMSE = 0.78).

In contrast, composite sampling in the high-prevalence setting did not demonstrate statistically significant associations at any lag. The DLM showed negligible explanatory power (adjusted R^2^ = −0.018), and similar results were observed in GLM and GAM, both of which showed no significant lag terms. Validation performance was also poor (r = −0.63; RMSE = 3.15), indicating limited predictive utility for this sampling approach in high-prevalence conditions. Detailed DLM estimates for the high-prevalence setting are presented in Table 2.

In the low-prevalence setting, composite sampling demonstrated the strongest and most consistent temporal association with reported COVID-19 cases. In the DLM, Lag 1 showed the strongest association (estimate = 3.833, *p* = 0.002), with an additional significant effect at Lag 0 (estimate = 2.503, *p* = 0.049), and good model fit (adjusted R^2^ = 0.48). These patterns were consistent across GLM and GAM analyses, with GAM indicating strong nonlinear support and high explanatory capacity. Although validation analysis showed moderate predictive performance, the agreement across multiple modeling approaches supports an approximate one-week lead relationship for composite sampling in low-prevalence conditions.

Grab sampling in the low-prevalence setting also demonstrated statistically significant associations, particularly at Lag 1 (estimate = 0.198, *p* = 0.006), with additional contributions from Lag 2. These findings were generally consistent across DLM, GLM, and GAM analyses. However, validation results indicated lower predictive stability (r = −0.45; RMSE = 5.43), suggesting that grab sampling is more sensitive to short-term variability and less stable compared to composite sampling in low-prevalence settings. Detailed DLM estimates for the low-prevalence setting are presented in Table 3.

While Table 2 and Table 3 present detailed DLM outputs, Table 4 provides an integrated summary of findings across modeling approaches, facilitating comparison of lag structures, model consistency, and predictive performance across sampling strategies and epidemiological settings. Detailed statistical outputs, including coefficient estimates, standard errors, and significance levels from all models (DLM, GLM, and GAM), are presented in Supplementary Table S1.

Across all settings, similar lag structures were identified using DLM, GLM, and GAM, indicating that the observed temporal relationships are not dependent on a single modeling framework. Grab sampling demonstrated the most consistent and stable lag structure in high-prevalence settings, whereas composite sampling showed stronger and more stable associations in low-prevalence settings. In contrast, composite sampling in high-prevalence conditions showed consistently limited performance across all models and validation metrics.

In addition to model consistency and lag structure, the magnitude of estimated effects provides further insight into the relationship between wastewater viral concentrations and reported case counts. The magnitude of the estimated effects indicates that increases in wastewater viral concentrations are associated with corresponding increases in reported case counts at specific time lags. Notably, larger effect sizes observed in low-prevalence settings suggest that wastewater signals may be more sensitive to changes in transmission dynamics when background incidence is low. However, effect sizes should be interpreted cautiously, as they are influenced by variability in sampling methods, population size, and underlying epidemiological conditions.

Overall, the results demonstrate that lag structure is both prevalence-dependent and sampling-method-dependent. Grab sampling was more informative in high-prevalence settings, where continuous viral shedding may enhance detection of short-term fluctuations, whereas composite sampling performed better in low-prevalence settings, where temporal aggregation improves detection of intermittent signals.

However, the estimated lead times should be interpreted cautiously, as they represent approximate temporal associations rather than precise predictive intervals. In addition, variability in validation performance indicates that wastewater signals are more suitable for identifying temporal trends than for generating precise predictions of case counts.

### 3.4. Variant Detection and Genomic Concordance

To track circulating SARS-CoV-2 variants in both high and low prevalence areas, we performed whole genome sequencing (WGS) using a targeted amplicon sequencing method with Oxford Nanopore Technologies on samples collected from these areas using composite and grab sampling methods. In total, 237 (68%) of the samples passed the sequence quality threshold with 60% coverage across the SARS-CoV-2 genome at a minimum depth of 10×, making them eligible for both the variant and lineage analysis; these samples were utilized in the Freyja_Plot analysis and Freyja_Dashboard visualization [35].

#### 3.4.1. High-Prevalence Area

**Figure 4:**
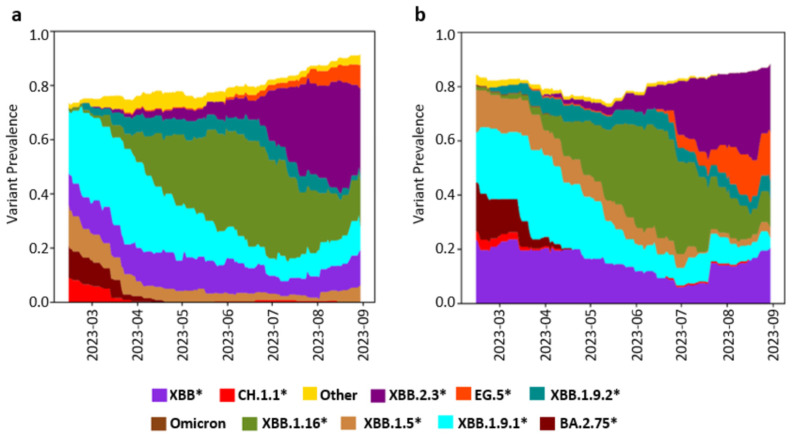
Temporal distribution of SARS-CoV-2 Omicron sublineages in wastewater in a high-prevalence area. The prevalence of circulating sublineages was estimated from samples collected between February and August 2023, using (**a**) grab sampling and (**b**) composite sampling. Variant proportions were derived from median estimates using Freyja (v1.4.9) and are color-coded. Lineages marked with an asterisk (e.g., XBB.1.5*, XBB.1.16*, XBB.2.3*) denote the respective lineage and all of its sublineages.

In the high-prevalence area (Figure 4), our study identified SARS-CoV-2 variants of concern (VOCs) from the Omicron sublineages BA.2.75, XBB, XBB.1.9.1, XBB.1.5, XBB.1.16, and XBB.2.3 as the primary circulating variants during the study period. During the early phase (February–April), three sublineages XBB.1.9.1, XBB, XBB.1.5, and BA.2.75 dominated, with XBB.1.9.1 being the most prevalent. In May, XBB.1.16 rose to dominance, displacing the previously dominant sublineages, while BA.2.75 disappeared entirely. XBB.1.16 maintained its dominance until August, when XBB.2.3 emerged as the main circulating variant and remained prevalent through the end of the study in September. Other lineages, including EG.5, XBB.1.9.2, and CH.1.1, were also detected but did not dominate during any specific month. Additionally, comparison of WGS results from samples collected using both sampling methods revealed consistent trends in variant circulation within the area.

#### 3.4.2. Low Prevalence Area

**Figure 5:**
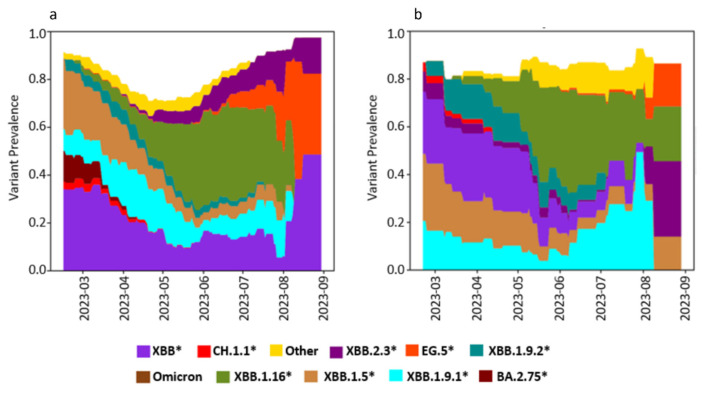
Temporal distribution of SARS-CoV-2 Omicron sublineages in wastewater in a low prevalence area. The prevalence of circulating sublineages was estimated from samples collected between February and August 2023, using (**a**) grab sampling and (**b**) composite sampling. Variant proportions were derived from median estimates using Freyja (v1.4.9) and are color-coded. Lineages marked with an asterisk (e.g., XBB.1.5*, XBB.1.16*, XBB.2.3*) denote the respective lineage and all of its sublineages.

In the low-prevalence area (Figure 5), the main circulating Omicron subvariants identified were XBB, XBB.1.5, XBB.1.9.1, XBB.1.16, XBB.2.3, and EG.5. Similar to the high-prevalence area, XBB, XBB.1.5, and XBB.1.9.1 were the predominant variants from March to April 2023. However, unlike the high-prevalence area, XBB.1.9.1 was not the most prevalent in the early period, with XBB and XBB.1.5 exhibiting higher dominance. Similar to the high-prevalence area, XBB.1.16 became the main circulating variant from May to August 2023. In August, the four main circulating subvariants were EG.5, XBB.2.3, XBB.1.16, and XBB.1.5.

Minor discrepancies were observed in the trends of SARS-CoV-2 variants circulating in the low-prevalence area between samples collected using composite and grab sampling methods. Notably, the BA.2.75 variant was detected only in grab samples, while the XBB.1.16 variant was not detected in grab samples collected toward the end of the study in August. Despite these differences, both sampling methods generally produced consistent trends in the low-prevalence area.

### 3.5. Comparison of Variant Trends Between Wastewater and Clinical Samples

**Figure 6:**
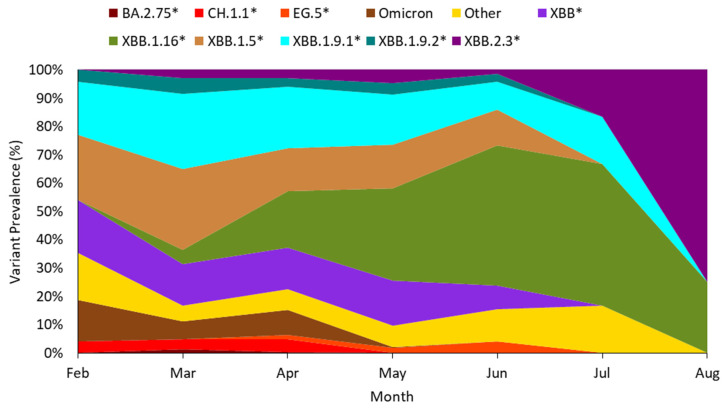
SARS-CoV-2 variants circulating in Selangor clinical samples from 6 February 2023 to 31 August 2023, based on data obtained from GISAID. Lineages marked with an asterisk (e.g., XBB.1.5*, XBB.1.16*, XBB.2.3*) denote the respective lineage and all of its sublineages.

To compare trends in SARS-CoV-2 variant circulation between WBS and clinical surveillance, we analyzed data from GISAID corresponding to our study period, spanning from 6 February 2023 to 31 August 2023 (Figure 6). A total of 737 SARS-CoV-2 sequences with lineage assignments were submitted from samples collected during this timeframe: 48 in February, 143 in March, 231 in April, 230 in May, 71 in June, 6 in July, and 8 in August. Consistent with WBS data, the predominant variants in February and March were XBB*, XBB.1.5*, and XBB.1.9.1*. Similarly, XBB.1.16* emerged in March and became one of the dominant variants from May through to the end of the study period. The main difference between WBS and clinical data was observed from July until August, when clinical data reported only two dominant sublineages XBB.2.3* and XBB.1.16*, while another two sublineages (XBB.1.5* and EG.5*), which were detected in WBS, were absent from the clinical report. However, the clinical data for this period included only 14 samples, which may introduce bias and limit reliability.

## 4. Discussion

### 4.1. Optimizing Wastewater Sampling Design for Early Warning Accuracy in High- and Low-Prevalence Settings

The findings suggest that the interaction between sampling strategy and community prevalence fundamentally influences detection performance, linking methodological approach with epidemiological context to shape the effectiveness of wastewater-based surveillance. This study demonstrates that grab and composite sampling perform differently depending on community prevalence. Grab sampling exhibited higher sensitivity and longer predictive lead times under high-prevalence conditions, likely because short-duration sampling captures transient viral peaks generated by continuous shedding. In contrast, composite sampling integrates over time and appears to perform more consistently when prevalence is low, as temporal accumulation increases the probability of detecting sporadic shedding events which require accumulation to reach detection thresholds. Our findings are consistent with Li et al.’s (2024) [19] findings about how sampling approach and infection prevalence interact to govern surveillance effectiveness in wastewater monitoring of SARS-CoV-2.

Quantitatively, grab sampling in high-prevalence areas demonstrated an approximate two-week lead association with clinical case surges, while composite sampling in low-prevalence areas showed the strongest temporal association at an approximate one-week lead. These findings define an early warning window of approximately 7–14 days consistent with global observations that wastewater viral concentrations typically precede reported cases and hospitalizations by one to two weeks [19,36]. Another study in the United States (2023–2024) reported that SARS-CoV-2 RNA concentrations in wastewater typically preceded hospital admissions by 2 to 12 days, while studies in Venezuela detected viral RNA approximately four to six days before official case reporting [7,17]. Unlike studies limited to persistent hotspots, this work demonstrates that such predictive capacity extends to low-prevalence communities in a tropical, middle-income context, underscoring the robustness of WBS across variable infrastructure and climatic conditions.

The consistency of key lag structures across DLM, GLM, and GAM supports the overall robustness of the main findings. However, the observed one- to two-week lead relationships should be interpreted as approximate and context-dependent rather than as precise predictive intervals. Differences in validation performance further indicate that wastewater signals are more informative for identifying temporal trends than for generating precise forecasts of case counts. In addition, the relatively limited effective sample size available for lag modeling (n = 26 weeks after accounting for lag structure) may have reduced statistical power and increased sensitivity to short-term temporal variability. Consequently, the predictive relationships identified in this study should be interpreted cautiously and may benefit from further validation using longer surveillance periods and additional sampling sites.

Lead detection in wastewater likely reflects a combination of factors, including variable viral shedding, sewer system transport, and the time over which samples are collected. This integration of factors determines how quickly changes in community infection are reflected in wastewater data [37]. Grab samples tend to capture short-lived concentration spikes, whereas composite samples average them out, attenuating the signal but improving detection stability at low incidence. The larger coefficients observed in the low-prevalence composite models suggest that temporally integrated sampling may enhance the detectability of intermittent shedding events when background transmission is low. In contrast, weaker or absent effects in the high-prevalence composite models suggest that temporal averaging may dilute short-term fluctuations that are better captured by grab sampling. The strength of the lagged associations observed in this study suggest that viral RNA concentrations in wastewater can act as a reliable leading indicator of community transmission intensity.

Lead times are inherently context-dependent and may also vary with behavioral patterns, sewer hydraulics, environmental factors, and clinical reporting delays [38,39,40,41,42,43,44,45]. Nevertheless, the consistency of predictive relationships across sites highlights the reproducibility of WBS as an epidemiological signal. Collectively, these results suggest that local infection prevalence is associated with the relative performance of sampling strategies. Optimal sampling strategy grab sampling is advantageous for rapid response in high-transmission settings, while composite sampling enhances detection reliability under low-incidence conditions. Tailoring sampling design to the epidemiological context may therefore improve the temporal fidelity and public health utility of wastewater surveillance systems.

### 4.2. Wastewater-Based Genomic Monitoring as a Complement to Clinical Sequencing

In Malaysia, the shift from mass population screening to self-testing using Rapid Test Kit Antigen (RTK-ag) has reduced the number of clinical diagnostic samples available for sequencing [46,47]. Maintaining genomic surveillance remains critical as emerging sublineages continue to pose risks. WBS offers a valuable complementary strategy, especially during periods of low clinical testing, to sustain SARS-CoV-2 genomic monitoring. Our study demonstrated that WBS can detect and track variant circulation in both high and low prevalence areas and its changes over time. In both areas, XBB-related sublineages (XBB*, XBB.1.5*, XBB1.9.1* and XBB.1.5*) were the dominant sublineages throughout the study period, consistent with the overall trend seen in clinical data in Selangor. This is consistent with other studies that showed wastewater genomic surveillance reliably mirrored the spread and shifting frequencies of the SARS-CoV-2 variants, matching results from clinical genomic surveillance [11,29,48,49].

During the period of reduced clinical data, differences between clinical genomic surveillance and WBS became more pronounced. Specifically, divergences emerged during July and August, when WBS detected additional sublineages XBB.1.5* and EG.5* that were not reported in clinical samples. This discrepancy likely stems from the limited number of clinical samples available during this period, which reduces the representativeness and statistical reliability of clinical surveillance. In contrast, WBS continuously aggregates signals from a broad population base and is not dependent on clinical testing rates, making it advantageous for genomic surveillance of SARS-CoV-2 when clinical testing is low.

### 4.3. Public Health Impact and Control Measures

As demonstrated globally and in this study for Malaysia, WBS can provide useful early indications of community transmission trends, with SARS-CoV-2 RNA in wastewater often detected approximately one to two weeks before increases in reported cases. These findings support the role of WBS as a complementary early warning component within public health surveillance systems. As demonstrated globally and in this study for Malaysia, WBS provides reliable early warnings by typically detecting SARS-CoV-2 RNA in wastewater one to two weeks before reported cases provide strong epidemiological evidence that WBS serves as an early indicator of community transmission. Significant lag associations across high- and low-prevalence areas demonstrate its sensitivity to changes in population viral load, while consistent detection of Omicron XBB sublineages and early identification of the XBB.1.5* and EG.5* variants highlight its genomic surveillance value. This role becomes increasingly vital given the decline in PCR testing rates and growing reliance on unreported self-administered antigen tests, which have reduced the availability of clinical specimens for sequencing.

This study enhances Malaysia’s national surveillance by providing unbiased, population-level indicators of infection trends independent of testing behavior, healthcare access, or reporting practices. As Malaysia transitions to the ‘Living with COVID-19’ phase, clinical focus has shifted from mass screening to targeted testing, and reductions in routine testing along with limited rapid antigen test availability in public facilities have contributed to substantial under-reporting. Statutory notifications now capture only professionally administered RTK or PCR-confirmed cases, reducing the reliability of case-based surveillance. These shifts highlight the value of WBS as a stable, consistent indicator of community transmission across both high- and low-prevalence settings, further strengthened by methodological advances such as genomic surveillance and standardized workflows.

Future expansion of WBS in Malaysia should emphasize integration with clinical, environmental, and meteorological datasets, in improving preparedness not only for SARS-CoV-2 but also for other pathogens such as influenza [50], enteric viruses [51], arboviruses [52], and antimicrobial-resistant bacteria, ultimately supporting a more responsive and data-driven public health system. Even with reduced clinical sample numbers, WBS in this study continued to reliably reflect infection and variant dynamics despite reduced clinical sample numbers, reinforcing its value as a resilient epidemiological indicator in contexts of constrained clinical surveillance.

### 4.4. Strength and Limitation

This study provides evidence showing that grab and composite sampling perform differently depending on community prevalence, offering practical guidance for optimizing wastewater surveillance strategies. The integration of digital PCR and genomic sequencing strengthens the reliability of detection and variant tracking during a period of reduced clinical testing. However, several limitations should be considered. The study was conducted at only two wastewater treatment plants within a single geographical region, which may limit generalizability to settings with different sewer infrastructure, population characteristics, and epidemiological conditions. In addition, reliance on routine case reporting and variable sequencing coverage may introduce bias in the comparison between wastewater and clinical data. Sequencing outputs may also be influenced by amplicon-based biases, including mutation-associated primer dropout. Furthermore, the absence of formal sensitivity or uncertainty analyses may limit the ability to fully assess the robustness and transferability of the observed associations; however, consistent patterns observed across multiple modeling approaches provide support for the stability of the findings.

Viral concentrations were not normalized using fecal indicators such as PMMoV. This was an intentional methodological choice to preserve direct comparability between sampling strategies, as the primary objective was to evaluate relative differences in detection performance under contrasting prevalence conditions. While normalization can improve comparability across systems, its effectiveness is context-dependent and may introduce additional variability, particularly in smaller or heterogeneous catchments where fecal indicators are influenced by site-specific factors. Previous studies have reported inconsistent relationships between fecal indicators and contributing population size, as well as mixed evidence regarding their ability to improve correlations between wastewater SARS-CoV-2 concentrations and clinical case data [53,54]. Importantly, the main findings of this study are based on relative comparisons (e.g., detection sensitivity and lag associations), which were consistent across multiple modeling approaches. However, the lack of normalization may limit direct comparison of absolute viral loads across different systems.

In future larger-scale surveillance programs, effective normalization may enhance inter-site comparability by accounting for variations in wastewater dilution, contributing population size, and sewer network characteristics, thereby enabling more reliable comparisons of viral signal magnitudes across diverse wastewater systems [55,56,57]. However, the effectiveness of normalization remains highly context-dependent. Inappropriate normalization approaches, particularly the use of certain physicochemical scaling factors in systems affected by substantial inflow and infiltration, may inadvertently introduce additional variability, amplify precipitation-driven dilution effects, and distort viral signal interpretation rather than improve comparability between sites [56,58].

## 5. Conclusions

This study demonstrates that WBS is a timely, sensitive, and complementary tool for infectious disease monitoring in Malaysia. Grab sampling was associated with stronger early wastewater signals in high-prevalence areas (approximate two-week lead association, *p* = 0.024), whereas composite sampling showed stronger temporal associations in low-prevalence settings (approximate one-week lead association, *p* = 0.002). Grab sampling captured stronger early signals in high-prevalence areas (two-week lead, *p* = 0.024), whereas composite sampling was more effective in low-prevalence settings (one-week lead, *p* = 0.002). Wastewater-based surveillance identified additional variant signals that were not captured in the available clinical sequencing dataset during the same period, including sublineages such as XBB.1.5* and EG.5*. These findings underscore the importance of tailoring sampling strategies to the epidemiological context to improve early detection capability and surveillance sensitivity. These findings underscore the importance of tailoring sampling strategies to the epidemiological context to maximize early detection and surveillance sensitivity. The observed patterns here provide quantitative guidance for optimizing WBS across diverse environmental and infrastructural settings, supporting proactive outbreak detection, targeted interventions, and real-time genomic monitoring to strengthen Malaysia’s preparedness for current and future infectious disease threats. However, these findings should be interpreted within the context of the limited number of study sites and warrant further validation across diverse wastewater systems and epidemiological settings.

## Figures and Tables

**Table 1:** Positivity rates of wastewater samples by sampling method and area prevalence, February–August 2023.

Area Prevalence	Sampling Method	Positive/Total	Positivity Rate (%)
Low	Composite	61/87	70.1
	Grab	80/87	92.0
High	Composite	85/87	97.7
	Grab	87/87	100.0
Overall	—	313/348	90.0

**Table 2:** Grab and composite sampling in high-prevalence areas.

Sampling Type	Term	Estimates	Std. Error	t-Value	*p*-Value
Grab	Intercepts	0.858	0.394	2.179	0.041 *
	Association				
	Lag 0 week	0.064	0.090	0.711	0.485
	Lag 1 week	0.070	0.097	0.724	0.477
	Lag 2 week	0.238	0.097	2.443	0.024 *
	Lag 3 week	−0.116	0.091	−1.282	0.214
Composite	Intercepts	1.616	1.016	1.590	0.127
	Association				
	Lag 0 week	0.332	0.390	0.852	0.404
	Lag 1 week	0.283	0.397	0.711	0.485
	Lag 2 week	0.309	0.396	0.779	0.444
	Lag 3 week	0.338	0.390	0.867	0.396

* *p*-value < 0.05, ** *p*-value < 0.01; high prevalence grab model fit: Residual SE = 1.146 (df = 21), R^2^ = 0.380, adjusted R^2^ = 0.262, F (4,21) = 3.221, *p* = 0.033; high prevalence composite model fit: Residual SE = 3.056 (df = 21), R^2^ = 0.145, adjusted R^2^ = −0.018, F (4,21) = 0.889, *p* = 0.488.

**Table 3:** Grab and composite sampling in low prevalence area.

Sampling Type	Term	Estimates	Std. Error	t-Value	*p*-Value
Grab	Intercepts	−0.757	1.493	−0.507	0.617
	Association				
	Lag 0 week	0.066	0.064	1.032	0.314
	Lag 1 week	0.198	0.064	3.089	0.006
	Lag 2 week	0.132	0.065	2.029	0.055 **
	Lag 3 week	0.115	0.066	1.750	0.095
Composite	Intercepts	1.578	1.204	1.311	0.204
	Association				
	Lag 0 week	2.503	1.200	2.085	0.049 *
	Lag 1 week	3.833	1.101	3.482	0.002 **
	Lag 2 week	1.737	1.109	1.566	0.132
	Lag 3 week	−0.448	1.212	−0.370	0.715

* *p*-value < 0.05, ** *p*-value < 0.01. Low prevalence grab model fit: Residual SE = 1.146 (df = 21), R^2^ = 0.380, adjusted R^2^ = 0.262, F (4,21) = 3.221, *p* = 0.033. Low prevalence composite model fit: Residual SE = 3.859 (df = 21), R^2^ = 0.560, adjusted R^2^ = 0.477, F (4,21) = 6.69, *p* = 0.001.

**Table 4:** Summary of lag associations, model consistency, and validation performance across sampling strategies and prevalence settings.

Setting	Sampling Method	Primary Lag Identified	Model Consistency (DLM/GLM/GAM)	Validation Performance (r/RMSE)	Overall Interpretation
High prevalence	Composite	None	No significant associations across all models	−0.63/High	Limited utility
	Grab	Lag 2	Consistent across DLM and GLM; supported by GAM	0.72/Low	Most informative and stable
Low prevalence	Composite	Lag 1 (primary), Lag 0	Strongly consistent across all models	−0.51/Moderate	Strong and stable
	Grab	Lag 1 (primary), Lag 2	Generally consistent but more variable	−0.45/Moderate–High	Moderate, less stable

## Data Availability

All raw wastewater sequencing data will be available via the NCBI Sequence Read Archive under the BioProject IDPRJNA1146140, https://www.ncbi.nlm.nih.gov/sra/PRJNA1146140 (accessed on 17 May 2026).

## Supplementary Materials

The following supporting information can be downloaded at: https://www.mdpi.com/article/10.3390/v18050583/s1, Table S1. Detailed results of DLM, GLM, GAM, and validation analyses across sampling strategies and prevalence settings.
