# Supplementary material for: Influence of Sampling Strategies and Disease Prevalence on SARS-CoV-2 Detection Dynamics in Wastewater Surveillance
Source: Viruses. 2026 May 21;18(5):583. doi: 10.3390/v18050583 (PMC13211533; doi:10.3390/v18050583)
Supplement: Supplementary file 1 [file viruses-18-00583-s001.zip › viruses-4200438-supplementary.pdf]

Table S1. Detailed results of DLM, GLM, GAM, and validation analyses across sampling strategies and prevalence settings.

| Setting | Sampling | Model | Lag | Estimate | Std. Error | *p*-value | Model Fit | Validation (r / RMSE) | Notes |
|---|---|---|---|---|---|---|---|---|---|
| High prevalence | Grab | DLM | Lag 2 | 0.238 | 0.097 | 0.024 | Adj $R^2 = 0.26$ | 0.72/0.78 | Significant lag |
| High prevalence | Grab | GLM | Lag 2 | NA | NA | 0.025 | NA | NA | Lag effect consistent with DLM |
| High prevalence | Grab | GAM | Lag 2 | NA | NA | 0.056 | Deviance explained = 34.6% | NA | Nonlinear support |
| High prevalence | Composite | DLM | All lags | NA | NA | >0.1 | Adj $R^2 = -0.018$ | −0.63/3.15 | No significant association |
| High prevalence | Composite | GLM | All lags | NA | NA | >0.1 | NA | NA | No significant association |
| High prevalence | Composite | GAM | All lags | NA | NA | >0.1 | Deviance explained = Low | NA | Weak model support |
| Low prevalence | Composite | DLM | Lag 1 | 3.833 | 1.101 | 0.002 | Adj $R^2 = 0.48$ | −0.51/4.57 | Strong association |
| Low prevalence | Composite | DLM | Lag 0 | 2.503 | 1.200 | 0.049 | Adj $R^2 = 0.48$ | −0.51/4.57 | Secondary effect |
| Low prevalence | Composite | GLM | Lag 1 | NA | NA | 0.003 | NA | NA | Consistent with DLM |

| | | | | | | | | | |
|---|---|---|---|---|---|---|---|---|---|
| Low prevalence | Composite | GAM | Lag 1 | NA | NA | 0.001 | Deviance explained = 72.9% | NA | Strong nonlinear support |
| Low prevalence | Grab | DLM | Lag 1 | 0.198 | 0.064 | 0.006 | Adj $R^2 = 0.26$ | −0.45/5.43 | Moderate association |
| Low prevalence | Grab | DLM | Lag 2 | 0.132 | 0.065 | 0.055 | Adj $R^2 = 0.26$ | −0.45/5.43 | Borderline significance |
| Low prevalence | Grab | GLM | Lag 1 | NA | NA | 0.008 | NA | NA | Consistent trend |
| Low prevalence | Grab | GAM | Lag 1 | NA | NA | 0.012 | Deviance explained = Moderate | NA | Nonlinear support |

**Notes:**

NA = not applicable

NS = not significant
